# Procainamide-facilitated induction of Purkinje premature ventricular contractions in a patient with idiopathic ventricular fibrillation

**DOI:** 10.1016/j.hrcr.2024.12.015

**Published:** 2025-01-02

**Authors:** Mai Badr, Sindhuja Palle, Craig Moskowitz, Aneesh Tolat

**Affiliations:** 1 Cardiovascular Division, Heart and Vascular Institute, Hartford Hospital, Hartford, Connecticut; 2 Section of Cardiac Electrophysiology, Heart and Vascular Institute, Hartford Hospital, Hartford, Connecticut

**Keywords:** Idiopathic VF, Short coupled-PVCs, PVC ablation, Electrophysiology study, Defibrillator

**Key Teaching Points**

• Idiopathic short coupled PVCs often arise from the Purkinje system. They can be associated with idiopathic VF and are a potential target for ablation.
• Previous literature has reported the use of ajmaline and IV flecainide for induction of short coupled PVCs implicated in idiopathic VF. However, these medications are not available in many countries.
• Intravenous procainamide may be useful for the induction of short coupled PVCs from the Purkinje system. This may then provide a target to guide successful catheter ablation of these PVCs.

## Introduction

Idiopathic ventricular fibrillation secondary to short coupled PVCs (Sc-PVCs) can be challenging to diagnose and treat given the need to exclude other forms of heart disease. In addition, the presentation of ventricular fibrillation (VF) often leads to a focus on stabilizing the patient followed by investigation into the potential explanations for the VF event. Sc-PVCs can be a target for ablation when found to trigger VF. They often arise from the Purkinje system and often have periods of relative quiescence. Recent publications have shown the use of sodium channel blockers flecainide and ajmaline to induce Purkinje Sc-PVCs during pharmacologic testing.^1^ However, intravenous (IV) flecainide and ajmaline are not available in many countries. We report on the use of IV procainamide that successfully induced Sc-PVCs and allowed for successful mapping and ablation of the Sc-PVC arising from the Purkinje system.

## History of presentation

A 32-year-old man presented with witnessed out-of-hospital cardiac arrest in 2013. He was found by emergency medical services to be in VF, and he received 3 external defibrillator shocks, with restoration of sinus rhythm. He has been treated with multiple antiarrhythmic medications including mexiletine, sotalol, and most recently quinidine (up to 648 mg/day) and verapamil (180 mg/day). He was implanted with a single-chamber implantable cardioverter defibrillator (ICD) and had undergone extensive testing. His echocardiogram and cardiac magnetic resonance imaging scan initially showed low normal left ventricular function, which has improved without delayed gadolinium enhancement. No significant prolongation of the QT interval was seen. Cardiac catheterization also was performed and did not show any significant obstructive coronary disease. Genetic testing also was performed and showed a variant of uncertain significance of the ANK2 gene.

**Figure 1:**
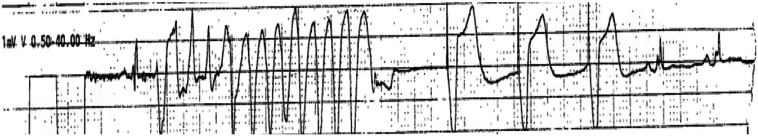
Premature ventricular complex inducing nonsustained ventricular tachycardia.

Over the years, the patient experienced periodic episodes of Sc-PVCs that triggered idiopathic VF, necessitating ICD shocks (Figure 1). The frequency of these episodes varied between 1 and 4 per year, with an average of 2.2 shocks for VF annually over a 10-year period. However, the total number of shocks administered for both VF and polymorphic ventricular tachycardia (VT) episodes ranged from 1 to 9 per year, averaging 3.7 shocks annually, with 6 shocks delivered for VF alone in the year preceding ablation. As a result, he was referred to the electrophysiology (EP) lab for outpatient Sc-PVC mapping and ablation.

**Figure 2:**
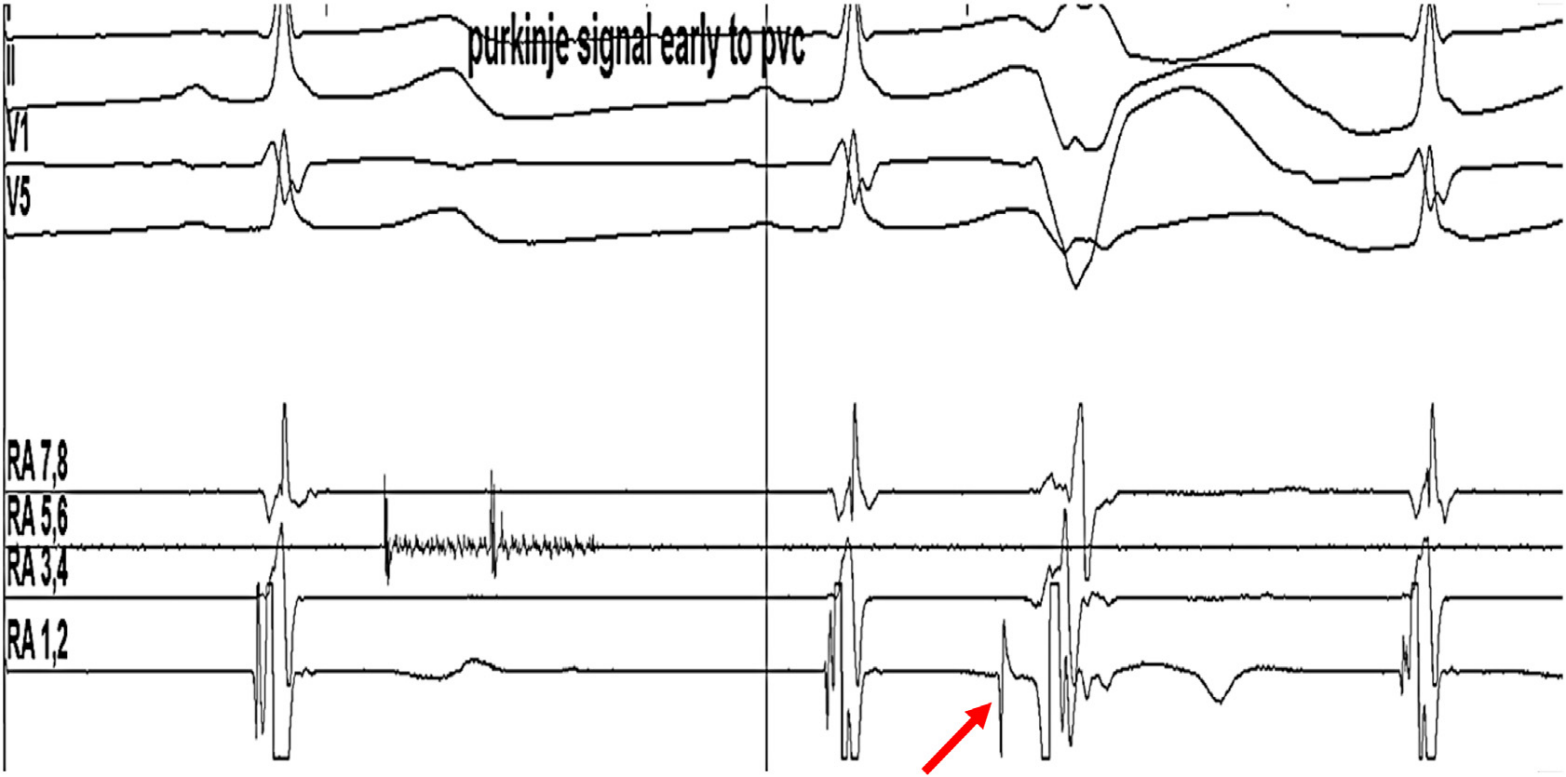
Intracardiac electrogram showing Purkinje signal (*red arrow*) early to PVC.

**Figure 3:**
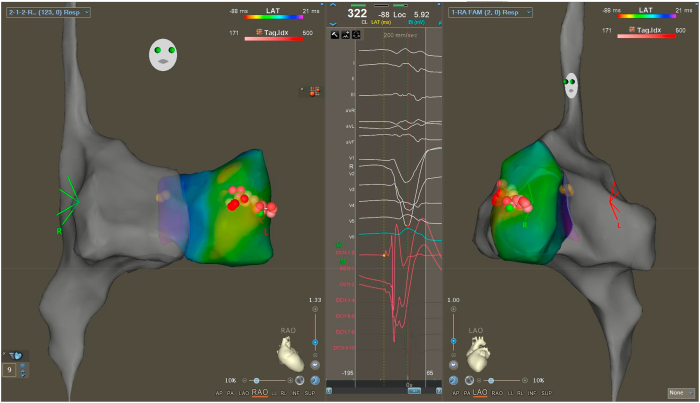
Premature ventricular complex (PVC) mapping. Ablation tags are shown in red, and His-bundle tags are shown in yellow.

On the morning of the scheduled procedure, the patient was not having PVCs before being brought into the EP lab. His quinidine, verapamil, and mexiletine were stopped 3 days earlier. After undergoing comprehensive diagnostic electrophysiology study, PVCs continued to remain absent under conscious sedation. Rapid atrial pacing, ventricular pacing, and isoproterenol infusion did not induce any ventricular arrhythmia. The patient’s sedation was stopped; however, he continued to remain free of PVCs. Intravenous procainamide (1 g over 30 minutes) was then given with real-time monitoring using the EP lab recording system and successfully induced monofocal PVCs after completion of the drug infusion (Figure 2) with left bundle branch block morphology. The coupling interval of the Sc-PVCs was 280 milliseconds, and they were previously absent and were occurring in trigeminy and quadrigeminy. Using a decapolar mapping catheter (Decanav, Biosense-Webster, Irvine, CA), PVCs were noted to be consistently early by 30 to 50 milliseconds in the area of the RV Purkinje fibers. No episodes of VT or VF were seen with these PVCs. Mapping of the earliest activation is shown in Figure 3. Radiofrequency ablation was performed with an irrigated tip Thermocool Smart touch SF catheter (Biosense Webster), using power of 25 to 30W. After ablation, the patient continued to have rare PVCs of a different morphology, but with marked decrease in frequency as compared with pre-ablation.

The patient was taken off Mexiletine, and continued verapamil and quinidine and did not have any episodes of VF for the next 14 months.

## Discussion

The diagnosis and management of idiopathic VF (IVF) remains challenging. Often periods of polymorphic VT and VF from Sc-PVCs are followed by relative quiescence for weeks and months. Once the diagnosis of IVF secondary to Sc-PVCs is made, management of the Sc-PVCs often involves suppression with medication or ablation. For catheter ablation to be successful, Sc-PVCs need to be identified during cardiac electrophysiology study, which often can be challenging. We report on the first known use of procainamide to facilitate Sc-PVC induction and catheter ablation of Purkinje PVCs.

IVF is identified as unexplained VF within a structurally normal heart with a negative comprehensive workup.^1^^,^^2^ In 6.6% to 17% of patients, IVF is preceded by a Sc-PVC,^3^ defined by a short coupling interval (<350 msec) from the preceding complex.^4^^,^^5^ Invasive mapping studies have identified the origin of Sc-PVCs as originating from the Purkinje system, making PVC ablation the gold standard approach for IVF caused by Sc-PVCs.^6^^,^^7^ Recenty, IV flecainide and ajmaline were identified during EP study to induce Sc-PVCs in patients being evaluated for Brugada pattern.^1^ However, IV flecainide and ajmaline are not available in the United States, and other alternatives need to be available for evaluation of such patients. Procainamide, also a sodium channel blocker, may be 1 such medication based on our observations in this case.

Administration of IV procainamide in this case was immediately associated with the induction of short coupled PVCs. Although use of medications to induce Sc-PVCs should be administered with caution, because they can also be associated with PMVT and VF, we did not observe this occurring in this case.

Our understanding of the mechanisms by which these medications work by favoring local reentry or afterdepolarizations is also limited.^7^ Quinidine is often used as an effective means for treatment of idiopathic VF, presumably by its impact on Ito current, whereas IV flecainide, ajmaline, and procainamide appear to potentiate Purkinje PVCs. Further work is required to better understand these observed differences in the action of these medications.

## Conclusions

In a patient with IVF related to Purkinje Sc-PVCs, procainamide was found to induce these PVCs, adding to evidence that sodium channel blockers may share a distinct pathophysiologic mechanism in these patients. This case highlights the possible utility of procainamide in inducing short-coupled PVCs to guide PVC ablation in the EP laboratory.
